# Neuropeptide S reduces duodenal bicarbonate secretion and ethanol-induced increases in duodenal motility in rats

**DOI:** 10.1371/journal.pone.0175312

**Published:** 2017-04-06

**Authors:** Wan Salman Wan Saudi, Markus Sjöblom

**Affiliations:** Department of Neuroscience, Division of Physiology, Uppsala University, Uppsala, Sweden; University of California Los Angeles, UNITED STATES

## Abstract

Alcohol disrupts the intestinal mucosal barrier by inducing metabolic and functional changes in epithelial cells. Recently, we showed that neuropeptide S (NPS) decreases duodenal motility and increases mucosal paracellular permeability, suggesting a role of NPS in the pathogenesis of disorders and dysfunctions in the small intestine. The aim of the present study was to investigate the effects of NPS on ethanol- and HCl-induced changes of duodenal mucosal barrier function and motility. Rats were anaesthetized with thiobarbiturate, and a 30-mm segment of the proximal duodenum with an intact blood supply was perfused *in situ*. The effects on duodenal bicarbonate secretion, the blood-to-lumen clearance of ^51^Cr-EDTA, motility and transepithelial net fluid flux were investigated. Intravenous (i.v.) administration of NPS significantly reduced duodenal mucosal bicarbonate secretion and stimulated mucosal transepithelial fluid absorption, mechanisms dependent on nitrergic signaling. NPS dose-dependently reduced ethanol-induced increases in duodenal motility. NPS (83 pmol·kg^-1^·min^-1^, i.v.) reduced the bicarbonate and fluid secretory response to luminal ethanol, whereas a 10-fold higher dose stimulated fluid secretion but did not influence bicarbonate secretion. In NPS-treated animals, duodenal perfusion of acid (pH 3) induced greater bicarbonate secretory rates than in controls. Pre-treating animals with Nω-nitro-L-arginine methyl ester (L-NAME) inhibited the effect of NPS on bicarbonate secretion. In response to luminal acid, NPS-treated animals had significantly higher paracellular permeability compared to controls, an effect that was abolished by L-NAME. Our findings demonstrate that NPS reduces basal and ethanol-induced increases in duodenal motility. In addition, NPS increases luminal alkalinization and mucosal permeability in response to luminal acid via mechanisms that are dependent on nitric oxide signaling. The data support a role for NPS in neurohumoral regulation of duodenal mucosal barrier function and motility.

## Introduction

Long-term and excessive consumption of alcohol is associated with the etiology of liver disease [1]. However, moderate intake of alcohol has been shown to be beneficial by lowering the risk of coronary heart disease [2, 3]. After intake, alcohol is absorbed by the epithelium in the foregut, mainly in the duodenum but also to some minor extent in the stomach [4, 5].

Previous experiments from our laboratory showed that perfusing the duodenal lumen with 15% ethanol in saline induces potent increases in mucosal paracellular permeability [6]. Interestingly, the neuroendocrine hormone melatonin was shown to reduce the ethanol-induced increases in duodenal paracellular permeability partly via an enteric neural pathway in a dose-dependent manner. Furthermore, melatonin was also shown to inhibit increases in duodenal motor activity in response to luminal ethanol [6, 7]. Although not fully described, it is plausible that the endogenous gastrointestinal neuroendocrine system may influence the magnitude of ethanol-induced changes to the regulation of tight junctional protein displacement and motility [6, 7].

Neuropeptide S (NPS), named after its serine residue, was recently shown to regulate duodenal motor activity and duodenal mucosal paracellular permeability [8]. In addition, previous studies have investigated the effects of NPS on regulating arousal, wakefulness and anxiety [9–12]. In the gastrointestinal tract, NPS receptors (NPSR) are expressed by epithelial cells, enteroendocrine cells, leukocytes and smooth muscle cells [13–16], suggesting a broad role in regulating intestinal functions. Furthermore, a polymorphism in the NPSR gene was found to be highly expressed in mucosal epithelial tissues in asthma and inflammatory bowel disease (IBD) patients [14, 17], suggesting a role of NPS in inflammation. However, the role of NPS/NPSR in the regulation of duodenal barrier function in response to alcohol- or acid-induced post-prandial mucosal stress is not well characterized.

The aim of the present study was to elucidate the effects of NPS on ethanol- and acid-induced changes of duodenal barrier function and motility in anesthetized rats *in vivo*. Duodenal bicarbonate secretion was determined by back titration of the perfusate, mucosal paracellular permeability was assessed by measuring the blood-to-lumen clearance of ^51^Cr-EDTA, and duodenal motor activity was determined by measuring changes in intraluminal pressure. Our results show that NPS significantly changed duodenal barrier function and motility in response to luminal ethanol and acid, suggesting a possible role of this peptide in the pathogenesis of intestinal functional and/or inflammatory reactions.

## Materials and methods

### Chemicals and drugs

^51^Chromium-labeled ethylenediaminetetraacetic acid (^51^Cr-EDTA) was purchased from Perkin Elmer Life Sciences (Boston, MA, USA). Bovine albumin (A2153), sodium chloride (NaCl), hydrochloric acid (HCl), potassium chloride (KCl), N^ω^-Nitro-L-arginine methyl ester hydrochloride (L-NAME), the anesthetic 5-ethyl-5-(1’-methyl-propyl)-2-thiobarbiturate (Inactin^®^) were purchased from Sigma-Aldrich (St. Louis, MO, USA). Parecoxib (Dynastat^®^, Pfizer, NY, USA) were obtained from Apoteket AB (Uppsala, Sweden). Ethanol 95.5 vol-% (Etax A) was purchased from Solveco Chemicals AB (Täby, Sweden). NPS was purchased from Bachem AG (Bubendorf, Switzerland).

### Ethics statement

All experiments were performed according to the Guide for the Care and Use of Laboratory Animals of the National Institutes of Health and approved by Uppsala Ethics Committee for Experiments with Animals (Permit number: C309/10).

### Animals

60 male Sprague-Dawley rats weighing 300–350 g were obtained from Taconic, Ejby, Denmark. The animals were maintained under standardized temperature and light conditions (12:12-h light-dark cycle; temperature, 21–22°C). The rats were acclimatized in the Animal Department for at least one week before experiments and were caged in groups of two or more with access to water and chow *ad libitum* (R36, Lantmännen, Kimstad, Sweden). The animals were fasted but had free access to tap water for 16 hours (overnight) before the experiments. Experiments were started by anesthetizing the animal around 8 am with Inactin^®^ 120 mg/kg body weight intraperitoneally. To minimize preoperative stress, anesthesia was performed within the Animal Department by the person who had previously handled the animals. Then, the rat was immediately transferred to the laboratory for surgical procedure.

### Surgical procedure

At the laboratory, the animals were tracheotomized with a tracheal tube to facilitate respiration, and the body temperature was maintained at 37–38°C throughout the experiments using a heating pad controlled by a rectal thermistor probe.

Two femoral arteries and femoral vein were catheterized with PE-50 polyethylene catheters (Becton, Dickinson & Co., Franklin Lakes, NJ, USA). For continuous recordings of the systemic arterial blood pressure, one of the arterial catheters containing 20 IU/ml heparin isotonic saline was connected to a transducer operating a PowerLab system (AD Instruments, Hastings, UK). The other arterial catheter was used for taking blood samples. The vein catheter was used for drug injections and for the infusion of saline and ^51^Cr-EDTA at a rate of 1.0 ml/h.

A laparotomy was performed, and the common bile duct was catheterized with a PE-10 polyethylene tubing close to its entrance into the duodenum (2–3 mm) to prevent pancreatico-biliary juice from entering the duodenum. Soft silicone tubing (Silastic^®^, Dow Corning, 1 mm ID) was introduced into the mouth, pushed gently along the esophagus, guided through the stomach and pylorus, and secured by ligatures 2–5 mm distal to the pylorus. PE-320 tubing was inserted into duodenum approximately 2.5–3.5 cm distal to the pylorus and secured by ligatures. The proximal duodenal tubing was connected to a peristaltic pump (Gilson minipuls 3, Villiers, Le Bel, France), and the segment was continuously perfused with a 154 mM sodium chloride solution (saline) at a rate of ~0.4 ml/min. To complete the surgery, the abdominal cavity was closed with sutures, and the wound was covered with plastic foil. Parecoxib i.v. injection 10 mg/kg was given 30 min after completion of the surgery to reverse the surgery-induced paralysis of the intestine [18–20]. After surgery, ~60 min was allowed for the cardiovascular, respiratory, and intestinal function to stabilize before the experiments were commenced.

### Measurement of luminal alkalinization

The rate of luminal alkalinization was determined by back titration of the perfusate to pH 4.90 with 10 mM HCl under continuous gassing (100% N_2_) using pH-stat equipment (Autoburette ABU 901 and pH-stat controller PHM 290, Radiometer, Copenhagen, Denmark). During experiments perfusing the luminal segment with HCl pH 3, the collected perfusate was back titrated to a pH of 2.95 using 50 mM HCl. The pH electrode was routinely calibrated with standard buffers before the start of the titration. The amount of titrated HCl was considered equivalent to the duodenal mucosal HCO_3_ˉ secretion. The rates of luminal alkalinization are expressed as micromoles of base secreted per centimeter of intestine per hour (μmol·cmˉ^1^·hˉ^1^).

### Measurement of duodenal mucosal permeability

After the completion of the surgery, ^51^Cr-EDTA was administered as an i.v. bolus of ~75 μCi followed by a continuous infusion at a rate of ~50 μCi per hour. The radioactive isotope was diluted in saline and infused at a rate of 1.0 ml/h. One hour was permitted for the tissue equilibration of the ^51^Cr-EDTA. Two blood samples (~0.2 ml each) were collected; the first was collected ten minutes before starting the experiment, and the second, after ending the experiment. The blood volume loss of the first blood sample was compensated by an injection of 0.2 ml of 7% bovine albumin solution. After centrifugation, 50 μl of the plasma was removed for measurements of radioactivity.

The duodenal segment was perfused with saline at a rate of 0.4 ml/min, and the perfusate was collected at 10-min samples. The luminal perfusate and blood plasma were analyzed for ^51^Cr-activity using a gamma counter (1282 Compugamma CS, Pharmacia, Uppsala, Sweden). A linear regression analysis of the plasma samples was made to obtain a corresponding plasma value for each perfusate sample. The clearance of ^51^Cr-EDTA from the blood to the lumen was calculated as described previously [21, 22] and expressed as (ml·minˉ^1^·100 gˉ^1^).

### Measurement of duodenal wall contractions

Measuring intraluminal pressure was used to assess the duodenal wall contractions. The inlet perfusion tubing was connected, via a T-tube to a pressure transducer, and intraluminal pressure was recorded on an IBM PC-compatible computer. The outlet tubing was positioned at the same level as the inlet tubing. An upward deflection of at least 2 mmHg above baseline was defined as a motor response. Changes in intraluminal pressure were recorded via a digitizer on a computer using PowerLab^®^ and the software Labchart7^®^ (ADInstruments Ldt. Hastings, East Sussex, UK). The duodenal motility was assessed in intervals of 10 min by planimetry to measure the total area under the pressure curve (area under the curve; AUC) during the sample period. The values given are mean ± SEM of three 10-min intervals (unless stated otherwise).

### Measurement of epithelial fluid flux

The difference in the weight of the collection vials with and without perfusate was used to measure flow over a 10-min interval. The perfusate volumes were determined after correcting for density for each solution. The density of the isotonic saline was arbitrarily set to 1.0. The duodenum was perfused (~0.4 ml/min) with isotonic saline or other solutions, and the perfusate was collected every 10 min. The net fluid flux across the duodenal mucosa was determined by subtracting the perfusate volume per 10 min from the peristaltic pump volume per 10 min, and the result is expressed as ml fluid per gram of wet tissue weight per hour (ml·g^-1^·h^-1^). The peristaltic pump volume was determined from the mean of two 10-min samples taken immediately after the termination of each experiment.

### Experimental protocol

In all experiments, the rates of duodenal bicarbonate secretion (μmol·cm^-1^·h^-1^), mucosal paracellular permeability (ml·min^-1^·100 g^-1^), motility (area under the curve; AUC) and net fluid flux (ml·g^-1^·h^-1^) as well as systemic arterial blood pressure (mmHg) and body temperature (°C) were monitored continuously and recorded at 10-min intervals.

Control experiments were performed by measuring the parameters above during a 150-min perfusion of isotonic saline (300 mOsm/kg) on the duodenal segment at a rate of ~0.4 ml/min.

In the animals exposed to luminal ethanol 15%, hydrochloric acid pH 3 or a combination of ethanol 15% and hydrochloric acid pH 3, the experiment started with perfusion of the duodenum with saline for 30 min to collect baseline data. Then, the duodenum was perfused for 30 min with ethanol 15%, hydrochloric acid pH 3 or the combination of ethanol 15% and hydrochloric acid pH 3 solutions.

For animals exposed to continuous i.v. NPS infusion, the experimental protocol was similar to the control experiment except that NPS was administered i.v. at 8, 83 and 833 pmol·kg^-1^·min^-1^ at 30, 70 and 110 min, respectively. Some groups of animals were pretreated 30 minutes before the experiment was started with an i.v. bolus of L-NAME (nitric oxide synthase, NOS inhibitor) 3 mg/kg followed by a 0.25 mg/h continuous i.v. infusion. In the latter group of animals, basal rates were recorded continuously 30 min prior administration of L-NAME.

In the animals pretreated with i.v. NPS and exposed to luminal ethanol 15%, the experiment protocol was similar to that for *animals exposed to luminal ethanol 15%* except that an NPS infusion was administered i.v. at 83 or 833 pmol·kg^-1^·min^-1^ a few minutes before the experiment commenced.

For animals pretreated with i.v. NPS and exposed to hydrochloric acid pH 3, the experiment protocol was exactly the same as for *animals exposed to luminal hydrochloric acid pH 3* except that an NPS infusion was administered i.v. at 83 pmol·kg^-1^·min^-1^ starting a few minutes before the experiment started. Some groups of animals were pretreated 30 minutes before experiment was started with an i.v. bolus of L-NAME (nitric oxide synthase, NOS inhibitor) 3 mg/kg followed by a 0.25 mg/h continuous i.v. infusion.

Immediately after finishing the experiment, the animal was euthanized with 1.0 ml bolus injection of 3 M KCl.

### Statistical analysis

Descriptive statistics are expressed as the mean ± SEM, with the number of experiments given in parentheses. The statistical significance of the data was tested by repeated measures analysis of variance (ANOVA). To test the differences within a group, a one-factor repeated measures ANOVA was used followed by a Tukey post-hoc test. Between groups, a two-way repeated measures ANOVA was used followed by a Bonferroni post-hoc test. Student’s paired t-test was used to test the differences within a group or between two groups. All statistical analyses were performed on an IBM-compatible computer using the Prism software package 6.05 (GraphPad Software Inc., San Diego, CA, USA). A *P* value less than 0.05 was considered significant.

## Results

**Fig 1:**
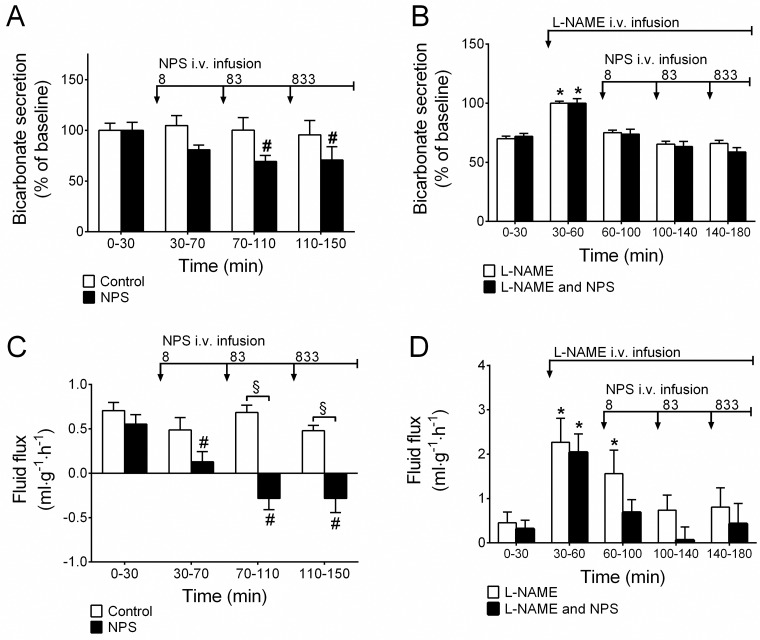
NPS reduces duodenal bicarbonate secretion and mucosal net fluid secretion, dependence on NO activity. **A)** Continuous i.v. infusion of NPS at 8–833 pmol·kg^-1^·min^-1^ reduced duodenal bicarbonate secretion and **C)** decreased duodenal mucosal net fluid secretion. **B & D)** Pre-treatment with L-NAME abolished the effects of NPS. # indicates a significant (*P*<0.05) reduction compared with baseline (0–30 min) in the same group. * indicates significantly (*P*<0.05) higher compared with baseline (0–30 min) in the same group. § indicates a significant (*P*<0.05) reduction compared to time-matched control animals.

In control animals (n = 9) where the duodenal segment was perfused with isotonic saline only, duodenal bicarbonate secretion, motility index and net fluid flux were stable at an average of 12.6±0.97 μmol·cm^-1^·h^-1^ (Fig 1A), 407±27 AUC/10 min (data not shown) and 0.58±0.15 ml·g^-1^·h^-1^ (Fig 1C), respectively, while duodenal mucosal paracellular permeability (blood-to-lumen clearance of ^51^Cr-EDTA) decreased modestly in a linear fashion from the start to the end of experiment (from 0.36±0.04 to 0.28±0.04 ml·min^-1^·100 g^-1^; *P*<0.05; data not shown). The mean arterial blood pressure and body temperature remained stable throughout the experiment in all of the groups (data not shown).

The effects of duodenal challenge with ethanol and acid have been demonstrated [6, 23]. It was shown that hydrochloric acid at a pH of 3 did not change the 15% ethanol-induced increases in duodenal bicarbonate secretion and mucosal paracellular permeability. In the present study we confirmed the previous results on bicarbonate secretion and permeability, but we also showed that HCl (pH 3) reduced 15% ethanol-induced increases in motility (n = 6, p<0.05, data not shown).

### Effects of i.v. NPS

Neuropeptide S has been shown to increase duodenal mucosal paracellular permeability and inhibit intestinal motility by mechanisms dependent on nitrergic signaling [8]. In the current study, NPS was demonstrated to reduce basal duodenal bicarbonate secretion (n = 11, p<0.05, Fig 1A) and decrease mucosal net fluid secretion (n, number of animals, N, number of observations, n/N = 11/99, p<0.05, Fig 1C). Pretreatment with an i.v. bolus of L-NAME 3 mg/kg followed by 0.25 mg/h continuous i.v. infusion induced transient increases in both bicarbonate secretion and net fluid flux and abolished the effect of NPS on bicarbonate secretion and net fluid flux (p>0.05, Fig 1B & 1D).

### Effects of i.v. NPS during luminal ethanol

**Fig 2:**
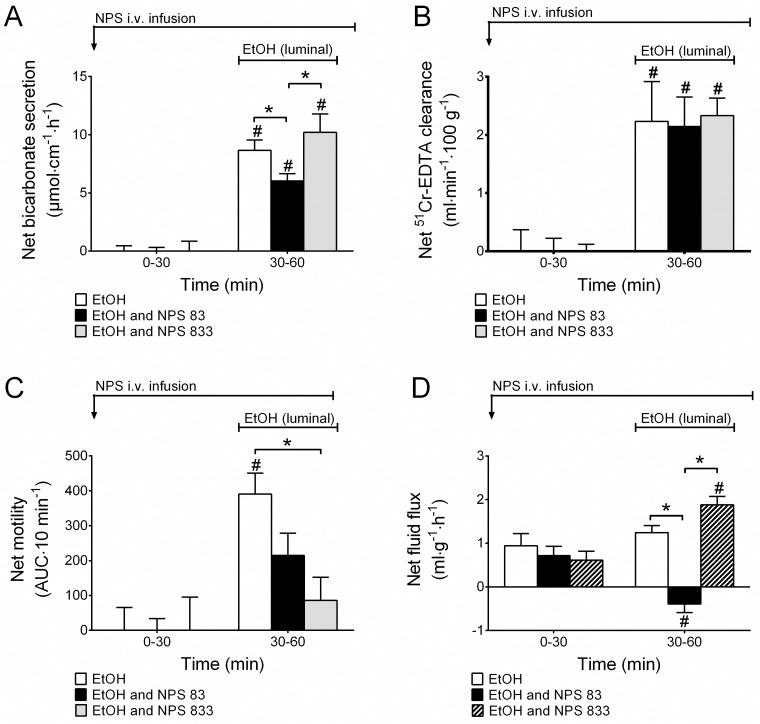
NPS reduces 15% EtOH-induced increases in duodenal motor activity. **A)** NPS at 83 pmol·kg^-1^·min^-1^ but not at 833 pmol·kg^-1^·min^-1^ decreases 15% EtOH-induced increases in duodenal bicarbonate secretion and **D)** epithelial net fluid secretion. **B)** NPS did not change 15% EtOH-induced increases in duodenal paracellular permeability, but **C)** it reduced 15% EtOH-induced increases in duodenal motility in a dose-dependent manner. # indicates a significant (*P*<0.05) increase compared with baseline (0–30 min) in the same group. * indicates significantly (*P*<0.05) lower than EtOH (and NPS 833 in A & D) -treated animals.

Administering a low concentration of NPS, 83 pmol·kg^-1^·min^-1^ i.v. (n = 6), during luminal perfusion of 15% ethanol significantly decreased the responses of duodenal bicarbonate secretion (p<0.05, Fig 2A) and epithelial net fluid secretion (p<0.05, Fig 2D) to ethanol. However, increasing the NPS infusion to 833 pmol·kg^-1^·min^-1^ (n = 7) did not have any effects on the bicarbonate or fluid flux responses to luminal ethanol.

**Fig 3:**
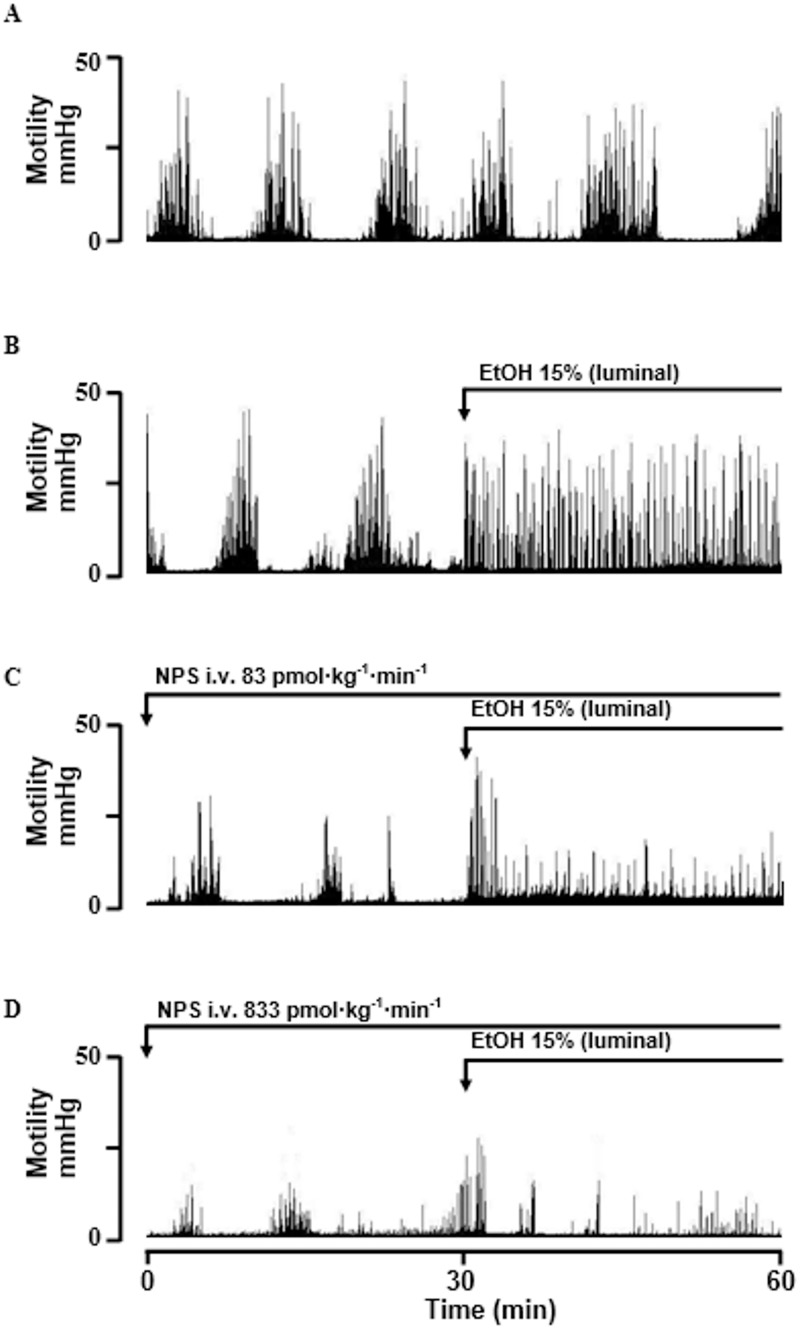
Representative experiment of NPS reducing 15% EtOH-induced increases in intraduodenal pressure (motility). All rats were pretreated with i.v. parecoxib 10 mg/kg approximately 60 min before the experiment started to reverse surgery-induced paralytic ileus. **A)** Saline perfusion with migrating motor complex (MMC) pattern. **B)** Luminal 15% EtOH increased motility. **C)** Continuous i.v. infusion of NPS reduced basal motility (time 0–30 min) and the motility effect of 15% EtOH. **D)** NPS at 10× concentration further decreased basal motility (time 0–30 min) and further dampened the effect of 15% EtOH.

NPS did not have any effects on ethanol-induced increases in duodenal mucosal paracellular permeability (Fig 2B), but it significantly reduced the ethanol-induced increases in duodenal motility dose-dependently (the net increases of EtOH, EtOH + NPS 83 and EtOH + NPS 833 pmol·kg^-1^·min^-1^ were 391±60, 214±64 and 85.86±66 AUC/10 min, respectively, p<0.05, Figs 2C & 3). As also shown previously [8], continuous i.v. infusion of NPS dose-dependently decreased basal duodenal motility, illustrated in Fig 3A–3D time 0–30 min. The motility during the basal period were in controls, NPS 83 and NPS 833 pmol·kg^-1^·min^-1^ 464±58, 197±49 and 145±51 AUC/10 min, respectively, p<0.05.

### Effects of i.v. NPS during luminal acid

**Fig 4:**
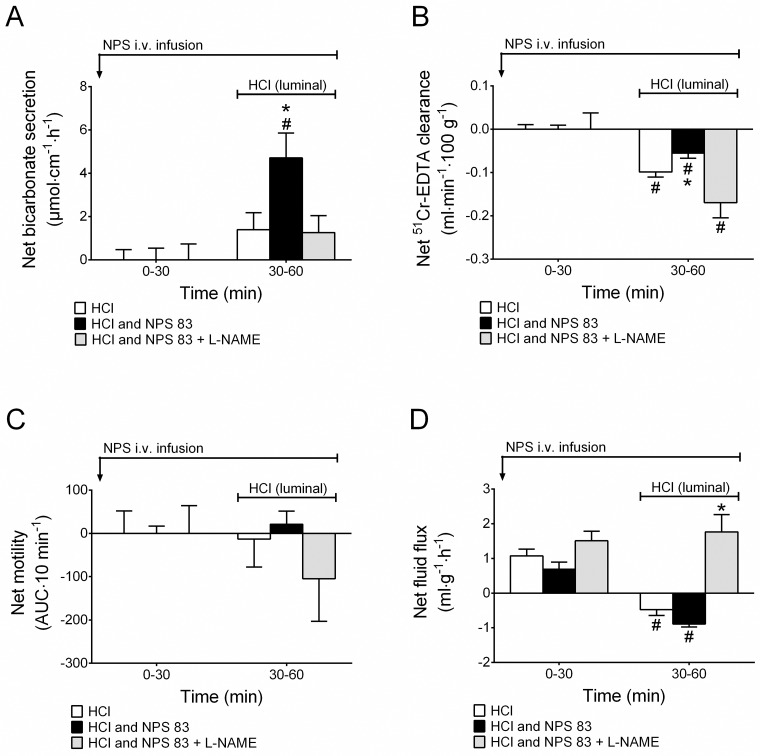
Co-administration of luminal hydrochloric acid 1.0 mM (pH 3) with intravenous NPS increases duodenal bicarbonate secretion and mucosal paracellular permeability in a NO-activity-dependent manner. **A)** Luminal HCl pH 3 alone had no effect, but co-administration with NPS continuous i.v. infusion 83 pmol·kg^-1^·min^-1^ significantly increased duodenal bicarbonate secretion and **B)** inhibited the duodenal mucosal paracellular permeability reduction except in animals pretreated with L-NAME. **C)** No significant changes were observed in duodenal motility. **D)** NPS had no effect on HCl-induced net fluid absorption. Pretreatment with L-NAME stimulated net fluid secretion. # indicates a significant (*P*<0.05) difference compared with baseline (0–30 min) in the same group. * indicates significantly (*P*<0.05) higher than the other groups.

Perfusing the duodenal lumen for 30 min with hydrochloric acid at a pH of 3 did not change the duodenal bicarbonate secretory rate or the mucosal paracellular permeability, similarly to what has been reported previously [23]. In addition, in the present study we show that duodenal motility also remained unchanged in response to a 30 min perfusion of hydrochloric acid (pH 3) (n = 7, Fig 4C). Intravenous NPS at 83 pmol·kg^-1^·min^-1^ (n = 7) significantly increased duodenal bicarbonate secretion (p<0.05), an effect that was abolished by pretreatment with 0.25 mg/h i.v. L-NAME infusion (n = 5, Fig 4A). NPS also significantly increased duodenal mucosal paracellular permeability, an effect that was abolished by L-NAME (Fig 4B). Furthermore, administration of i.v. NPS during luminal acid perfusion did not change the motility response to luminal acid (p>0.05, Fig 4C). Similarly, 83 pmol·kg^-1^·min^-1^ NPS did not change the net fluid flux (p>0.05, n/N = 7/63) response to luminal acid compared to luminal acid alone (n/N = 6/54). During pretreatment with L-NAME (n/N = 6/54), acid together with NPS did not induce net fluid absorption, instead net fluid secretion was observed (Fig 4D).

## Discussion

The etiology of functional gastrointestinal disorders and inflammatory bowel syndrome is incompletely understood, but it is suggested to involve dysregulation of the enteric neurohumoral control of intestinal mucosal barrier function and motility. In addition, it is widely accepted that these disturbances have multifactorial causation with both internal and external factors playing some role, and new findings within this area are important to better understand the causes of these disorders. We showed recently that neuropeptide S, a fairly newly described signaling molecule, depresses intestinal motility in humans as well as in rats [8]. It was also shown that NPS receptor subtype NPSR1 is highly expressed within the enteric nervous system and to a lesser extent on duodenal enterocytes. The finding that NPS induces increases in duodenal mucosal paracellular permeability makes this peptide an interesting candidate for further investigation of its role in the regulation of intestinal function.

In the present study we show that increased duodenal motility induced experimentally by luminal perfusion of moderate-concentration ethanol is markedly inhibited by the administration of NPS. Furthermore, NPS was also shown to significantly reduce basal duodenal mucosal bicarbonate secretion while stimulating fluid absorption.

A likely cause of abdominal symptoms and distention in patients with irritable bowel syndrome, abdominal bloating, or functional dyspepsia is depressed clearance of intestinal gas caused by intestinal dysmotility. In the duodenal segment, great amounts of gastric acid are mixed with bicarbonate originating from the pancreas and the duodenal mucosa, generating large quantities of CO_2_. Increased gas load or disturbed regulation of intestinal motility may cause distention and the sensation of discomfort.

Increased duodenal motility in response to luminal ethanol seems important for removing the irritant from the duodenal segment. An interesting observation of the present study was that the administration of NPS caused dose-dependent inhibition of the ethanol-induced increases in duodenal motility. The doses of NPS used in the present study are estimated to generate plasma concentrations from 0.5 to 50 nM, which are close to the EC_50_ of the normal NPSR1 (gene107^Asn^, ~32 nM) [24]. The effects of NPS on intestinal motility support previous findings that NPS reduces basal motility in both rats and humans [8]. It may also be speculated that a delayed, or disturbed, duodenal motility response to luminal ethanol would increase the risk of epithelial damage and disturbed mucosal barrier function as observed in patients with chronic alcoholism [25, 26]. It was shown previously that acid at a pH of 3 does not change basal motility. In response to perfusing the duodenal segment with HCl (pH 3) NPS also left duodenal motility unchanged.

Previous findings in mice have shown that central nervous, but not intraperitoneal, administration of NPS dose-dependently inhibits distal colonic transit [27] as well as restraint stress and corticotropin releasing factor-induced defecation [28]. A relevant question is whether NPS administrated intravenously, as in this study, cross the blood-brain barrier and exert its actions via central mechanisms rather than at the intestinal level. However, this would seem unlikely to us. Firstly, peptides of the molecular size of NPS cross the BBB poorly. Secondly, the origin of the motility stimulation are different in the present *in vivo* experiments and in the studies by Han *et al* and Petrella *et al*. Thirdly, these authors clearly separate the effects of NPS after central and intraperitoneal administrationusing doses of NPS close to those in the present study. However, our experiments were not designed to elucidate this and the interrelationship between centrally and enteric role of NPS in the regulation of gastrointestinal motility is an area of great interest and therefore warrants further studies.

It has been reported that low concentrations of ethanol induce non-cytotoxic effects on the intestinal mucosa by increasing the structural opening of the tight junctional protein zona occludin 1 (ZO-1) via myosin light chain kinase (MLCK) activation [29]. In a previous study we investigated the role of intestinal melatonin in ethanol-induced increases in duodenal mucosal paracellular permeability. It was shown that melatonin significantly reduced ethanol-induced increases in permeability by a mechanism involving nicotinic receptors in the enteric nervous system [6, 7]. Because NPS was suggested to potentially be involved in the pathogenesis of inflammatory diseases, such as in asthma and IBD [14, 17], we became interested in the role of NPS in paracellular permeability changes during ethanol perfusion. NPS is known to increase basal duodenal mucosal permeability [8]. In the present study, NPS was found not to have a significant role in ethanol-induced increases in permeability. However, it was shown that luminal acid reduces duodenal permeability, an effect that was inhibited by NPS infusion. The NPS-induced inhibition was abolished by pre-treatment with L-NAME, suggesting a mechanism involving NO signaling. To break the duodenal barrier, i.e., observe great increases in paracellular permeability, we have shown previously that acidity levels close to pH 1 are needed [6, 23], but such strong acidity was not tested in the present study.

Duodenal mucosal bicarbonate secretion is under central as well as local intestinal neurohumoral regulation. Isenberg *et al* showed in 1987 that patients with duodenal ulcer disease have a depressed basal secretory rate and a markedly reduced secretory response to luminal acid [30]. The mechanisms by which this secretion is regulated have been studied in detail over a long period of time, but novel findings are still made that are important for understanding how this secretion is controlled [31]. In the present study, we showed that NPS significantly decreased the basal bicarbonate secretory rate. The inhibitory effect of NPS was abolished by pre-treating the animals with the unselective NOS inhibitor L-NAME. NPSR1 is highly expressed in the myenteric plexus [8] and also on enteroendocrine cells [13], suggesting that the effects of NPS on bicarbonate secretion are mediated by the enteric nervous system. The finding that NPSR1 and nNOS are co-localized in neurons of the myenteric plexus further supports this hypothesis [8]. However, mucosal permeability and ion transport are mainly regulated by direct epithelial mechanisms and/or by neurons in the submucosal plexus. Since the myenteric and submucosal plexuses are highly integrated by interneurons, it is likely that changed neuronal activity in the myenteric neuronal network also affect mucosal permeability, fluid and ion secretions. In response to ethanol the administration of NPS had dual effects on the secretory rate. A low concentration of NPS reduced the bicarbonate secretory rate in response to ethanol, whereas increasing the NPS dose ten times did not have any effect on secretion.

Another interesting observation was that the secretory response to luminal acid was greater in animals treated with NPS compared to control animals. The effects of NPS on acid-induced secretion were significantly reduced by pre-treating the animals with L-NAME. It is evident that increased levels of prostaglandin E2 acting on the EP3 receptor subtype play a critical role in the duodenal secretory response to mucosal acidification [32]. However, whether NPS really increases the release of prostaglandin E2, or other prostanoids, needs further investigation. It is difficult to explain the finding that NPS reduces basal bicarbonate secretion but increases the secretory response to acid. One possibility is that the proinflammatory effects of NPS together with the acid exposure cause greater increases in the generation of prostaglandin E2 than occur in controls, whereas the secretory decrease observed during NPS treatment alone is mainly caused by reduced intestinal motility.

NPS also induced an absorptive effect on the duodenal epithelium, a mechanism dependent on NO. This observation is supported by others demonstrating that NO induces intestinal fluid absorption [33–36]. A role of NO is further supported by the fact that L-NAME abolished the absorption mediated by NPS. In contrast, NO has also been shown to be involved in stimulating fluid secretion [37, 38]. Mourad *et al* suggest that NO at physiological concentrations promotes fluid absorption, but during pathological conditions when NO is produced in greater amounts it facilitates fluid secretion [39]. This proposal is supported by our findings during ethanol perfusion, wherein NPS in a low concentration induced fluid absorption and reduced bicarbonate secretion while higher doses of NPS induced increases in both fluid and bicarbonate secretion.

In summary, the administration of NPS was found to reduce basal and ethanol-induced increases in duodenal motility. It was also shown that NPS reduces bicarbonate secretion and stimulates fluid absorption by mechanisms involving NO signaling. However, additional studies are needed to better understand the mechanism by which NPS regulates intestinal barrier function and motility. An interesting future objective would be to elucidate the effect of long-term administration, or endogenous over-production, of NPS and its involvement in the pathogenesis of intestinal dysmotility and suppressed barrier function.
